# Voltammetric Determination of 5-Hydroxymethyl-2-furfural in Processed Cheese Using an Easy-Made and Economic Integrated 3D Graphene-like Electrode

**DOI:** 10.3390/s22010064

**Published:** 2021-12-23

**Authors:** Yuzhen Li, Juanhua Zhang, Mengxiao Lv, Yihui Bai, Xuexiang Weng, Chunping You, Zhenmin Liu

**Affiliations:** 1 State Key Laboratory of Dairy Biotechnology, Shanghai Engineering Research Center of Dairy Biotechnology, Postdoctoral Workstation of Bright Dairy-Shanghai Jiao Tong University, Dairy Research Institute, Bright Dairy & Food Co., Ltd., Shanghai 200436, China; liyuzhen@brightdairy.com (Y.L.); liuzhenmin@brightdairy.com (Z.L.); 2 Key Laboratory of the Ministry of Education for Advanced Catalysis Materials, College of Chemistry and Life Sciences, Zhejiang Normal University, Jinhua 321004, China; juanhuazhang2021@163.com (J.Z.); lvmengxiao@zjnu.edu.cn (M.L.); baiyihui@zjnu.cn (Y.B.); xuexian@zjnu.cn (X.W.); 3 Synergetic Innovation Center of Food Safety and Nutrition, Jiangnan University, Wuxi 214122, China

**Keywords:** 5-hydroxymethyl-2-furfural, pencil graphite electrode, three-dimensional graphene-like surface, sensor, processed cheese

## Abstract

The concentration of 5-hydroxymethyl-2-furfural (HMF) is an important quality-related index in milk and milk products. Fast, cost-effective and environmentally friendly determination of HMF is of great significance in milk products control. In this study, a three-dimensional (3D) graphene-like surface (3DGrls) was successfully prepared within 5 min by an electrochemical amperometric pretreatment on a pencil graphite electrode (PGE). The fast-obtained 3D graphene-like surface increased the electrode surface area and enhanced the electron transfer capability without the addition of any harmful chemicals. The morphology and chemical composition of the obtained electrode were characterized by scanning electron microscope (SEM), X-ray photoelectron spectroscopy (XPS), Raman spectroscopy and electrochemical impedance spectroscopy (EIS). The results found that the electrochemical response to HMF at the prepared 3DGrls/PGE was 34 times higher than that at PGE. The modified electrode showed a good linear response to HMF in a concentration range of 0.35~116 μM with a low limit of detection (LOD) of 0.099 μM. The integrated electrode also exhibited excellent stability and wonderful antifouling property. Furthermore, the 3DGrls/PGE was successfully applied for the determination of HMF in three processed cheese samples with satisfactory results.

## 1. Introduction

5-hydroxymethyl-2-furfural (HMF) is a furan derivative, mainly used as a renewable material and a versatile platform molecule. It is also widely used in medicine, chemical industry, pesticide and other fields [1]. HMF is also produced in foods (milk, cheese, meat, bread, etc.) during heat treatment and long-term storage [2,3,4,5]. Due to the abundant carbohydrates and proteins, four different furfural compounds (HMF, furfural, furylmethylcetone and methylfurfural) were mainly produced by Maillard reaction between reducing sugars and lysine-rich proteins in milk products (liquid milk, milk powder, cheese, etc.). The concentration of HMF was found to be much higher than the other three [6,7]. Therefore, the concentration of HMF is an important quality-related index in milk products. However, the human central nervous system, liver, kidney and heart will be damaged when the concentration of HMF exceeds a certain limit of the body absorption [8]. Therefore, it is very important to establish a convenient, reliable and efficient method for the fast determination of HMF in milk products.

The common detection method for HMF is chromatography, such as high-performance liquid chromatography (HPLC) [9,10,11,12,13,14], headspace gas chromatography [15] and gas chromatography coupled with triple quadrupole mass spectrometry (GC-MS/MS) [16]. In addition, UV–Vis spectrophotometric methods are also official methods for HMF determination in different matrices [17,18,19]. However, all of these methods have some drawbacks, such as expensive and sophisticated equipment, toxic reagents and heavy interfering effects. In contrast, the electrochemical method has the advantages of simple operation, easy portability, short analysis time and high sensitivity, which can overcome the aforementioned problems [20]. Nanomaterials, especially two-dimensional (2D)-layered nanomaterials, have been widely used in high-efficiency electrochemical sensors because of their large specific surface area, excellent adsorption capacity, superior electrochemical activity and excellent signal amplification performance [21,22,23,24]. However, there are few reports on the quantitative determination of HMF in real samples by the electrochemical method, especially based on electrodes modified with nanomaterials [25,26,27,28,29]. Very recently, we reported the successful determination of HMF in milk by a black phosphorene nanosheets (BP)-modified glassy carbon electrode (GCE) [30]. Although this electrochemical method is simple and sensitive, the drop-casting preparation of BP on GCE leads to instability of the sensing signal. How to realize the detection of HMF in dairy products more stably, quickly and environmentally friendly is still an urgent problem to be solved in the dairy industry.

Graphene, a 2D carbon nanomaterial with a sp^2^-hybridised honeycomb structure, is an ideal material for electrochemical sensors due to its large surface area and fast electron transfer ability [31,32,33,34,35]. Three-dimensional (3D) graphene is one of the derivatives of graphene, which not only possesses the intrinsic properties of graphene but also has an interconnected 3D conductive network and special microenvironment structure [36]. Due to its large surface area, good diffusion performance and high electric conductivities, 3D graphene synthesized from various strategies has attracted increasing attention in sensor-related devices [37]. However, it has not been reported in HMF detection.

Up to now, the preparation methods of 3D graphene mainly include the electrochemical method [38], self-assembled method [39] and template-assisted method [40]. The main disadvantage of the electrochemical method is the uncontrollable morphology. The template-assisted chemical vapor deposition (CVD) method is commonly used to obtain 3D graphene with controllable morphology. However, many defects are generated during the process of removing the template, which affects the sensor performance [41]. The self-assembly method can realize the mass production of graphene with a low cost; however, its application is limited by defects such as a long preparation time, poor electrical conductivity and uncontrollable spatial network structure. The traditional fabrication of a 3D graphene-modified electrode usually involves first synthesizing the material offline and then immobilizing the material on the electrode surface using the drop-casting method. The two-step strategy has some problems, such as complicated synthesis and poor electrode response stability and reproducibility.

Recently, Parvez et al. [42] reported a method for electrochemically stripping graphite into graphene by applying a +10-V direct current (DC) voltage to an inorganic salt electrolyte. Inspired by the above work and the existing achievements of our research group [43], herein, a 3D graphene-like surface (3DGrls) was formed within 300 s in a Na_2_SO_4_ solution using the in situ electrochemical expansion of a pencil graphite electrode (PGE) without anything hazardous added. The ultrafast-prepared electrode, namely, 3DGrls/PGE, not only has a larger surface area but also enhances the electron transfer ability. Under the optimized conditions, the 3DGrls/PGE was used for the determination of HMF by linear sweep voltammetry (LSV) with a low detection limit and a wide linear range. Moreover, the 3DGrls/PGE was successfully applied to the direct determination of HMF in processed cheese.

## 2. Materials and Methods

### 2.1. Chemicals and Reagents

PGEs (2B, 0.5 mm in diameter) were purchased from Mitsubishi Pencil Company in Japan. Sodium sulfate (AR, ≥99%, Sinopharm Chemical Reagent Co. Ltd., Shanghai, China)), disodium hydrogen phosphate (AR, ≥99%, Sinopharm Chemical Reagent Co. Ltd., Shanghai, China), sodium dihydrogen phosphate (AR, ≥99%, Sinopharm Chemical Reagent Co. Ltd., Shanghai, China), potassium ferricyanide (AR, ≥99%, Sinopharm Chemical Reagent Co. Ltd., Shanghai, China), potassium ferrocyanide (AR, ≥99%, Sinopharm Chemical Reagent Co. Ltd., Shanghai, China), potassium chloride (AR, ≥99%, Sinopharm Chemical Reagent Co. Ltd., Shanghai, China, calcium chloride (AR, ≥93%, Sinopharm Chemical Reagent Co. Ltd., Shanghai, China), zinc chloride (AR, ≥99%, Sinopharm Chemical Reagent Co. Ltd., Shanghai, China), glucose (AR, ≥99%, Sinopharm Chemical Reagent Co. Ltd., Shanghai, China) and oxalic acid (AR, ≥99.5%, Sinopharm Chemical Reagent Co. Ltd., Shanghai, China) were used in the research..HMF (AR, ≥99%, Aladdin Chemistry Co. Ltd., Shanghai, China), lactose (AR, ≥98%, Aladdin Chemistry Co. Ltd., Shanghai, China), trichloroacetic acid (AR, ≥99%, Aladdin Chemistry Co. Ltd., Shanghai, China), ascorbic acid (AR, ≥99.7%, Aladdin Chemistry Co. Ltd., Shanghai, China) and L-lysine (AR, ≥99%, Aladdin Chemistry Co. Ltd., Shanghai, China) were used directly after purchase without further purification. The 0.1-M phosphate buffer solutions (PBS) (pH 9.18) were prepared by mixing NaH_2_PO_4_ and Na_2_HPO_4_ as the supporting electrolyte, and the pH was adjusted by NaOH. The solution of HMF was prepared in PBS (pH 9.18) and kept away from light. Ultrapure fresh water used in all runs, with a resistivity of 18 MΩ cm, was obtained from a Millipore water purification system (MilliQ, Molsheim, France).

### 2.2. Apparatus

All electrochemical measurements were performed using a CHI 660C electrochemical workstation (Chenhua Instrument Company, Shanghai, China) in a standard three-electrode cell system, consisting of a PGE or a modified PGE as the working electrode, a saturated calomel electrode (SCE) as the reference electrode and a platinum wire as the counter electrode. In the sensing of HMF, high-purity nitrogen gas was used to purify the solution for 10 min before running voltammetry experiments and was kept flowing above the solution to maintain the nonoxygen environments. EIS was obtained from the IM6E impedance measurement unit (Zahner, Germany). The 5-mM [Fe(CN)_6_]^3−/4−^ solution containing 0.1-M KCl was supported at an open circuit potential with an amplitude of 50 mV and a frequency range from 0.01 Hz to 100 kHz.

The morphology of the modified electrode was obtained from a Hitachi S-4800 scanning electron microscopy (SEM; Tokyo, Japan). The composition evidence was further inferred by X-ray photoelectron spectroscopy (XPS) using a K-Alpha XPS spectrometer (SCIENTIFIC ESCALAB 250) with Al-Kα X-ray radiation (1486.6 eV) as the excitation source. The X-ray diffraction (XRD) analysis of Bruker D8 ADVANCE in Germany was carried out with a graphite monochromatized Cu Kα radiation source (λ = 1.54056 Å). Raman spectra were obtained by a He/Ne laser at 633 nm on the Renishaw RM1000 spectrometer.

### 2.3. Fabrication of 3DGrls/PGE

The preparation of 3DGrls/PGE was based on our previous report [43]. In detail, the commercial PGE was sealed with AB glue in the middle, leaving the bottom end about 0.5-cm exposed to obtain a fixed geometric area of the electrode surface (about 0.73 cm^2^). The PGE was electrolyzed in 0.1-M Na_2_SO_4_ solution at a constant potential of 3.0 V (vs. saturated calomel electrode, SCE) for 300 s to acquire a 3D graphene-like surface. Then, the resulting electrode (denoted as 3DGrls/PGE) was cleaned with deionized water and dried at room temperature for further characterization.

### 2.4. LSV Determination of Real Samples

Three processed cheese samples were purchased from a local supermarket. A small amount (0.50 g) of crushed cheese was dispersed in 12 mL of water and sonicated for 30 min. After shaking for 20 min, the dispersion was heated in bath water (100 °C) for 25 min upon the addition of 5 mL of 0.15-M fresh oxalic acid. The cooling solution was then pretreated according to our previous report [44,45]. For LSV determination, a certain volume of the pretreated sample was added into the electrochemical cell, and PBS (pH 9.18) was added to give a final 10-mL solution. The standard curve was further used to calculate the content of HMF, and the results were compared simultaneously with HPLC results.

### 2.5. HPLC Determination of Real Samples

The HMF was determined by RP-HPLC-DAD, with a slight modification of the Albala-Hurtado method [46]. The 0.05 g of crushed cheese was dispersed in 12 mL of water and treated with ultrasound for 30 min. After shaking for 20 min, the dispersion was then pretreated according to our previous report [6].

## 3. Results and Discussion

### 3.1. Optimization of Preparation Conditions of 3DGrls/PGE

#### 3.1.1. Electrolyte Concentration

**Figure 1:**
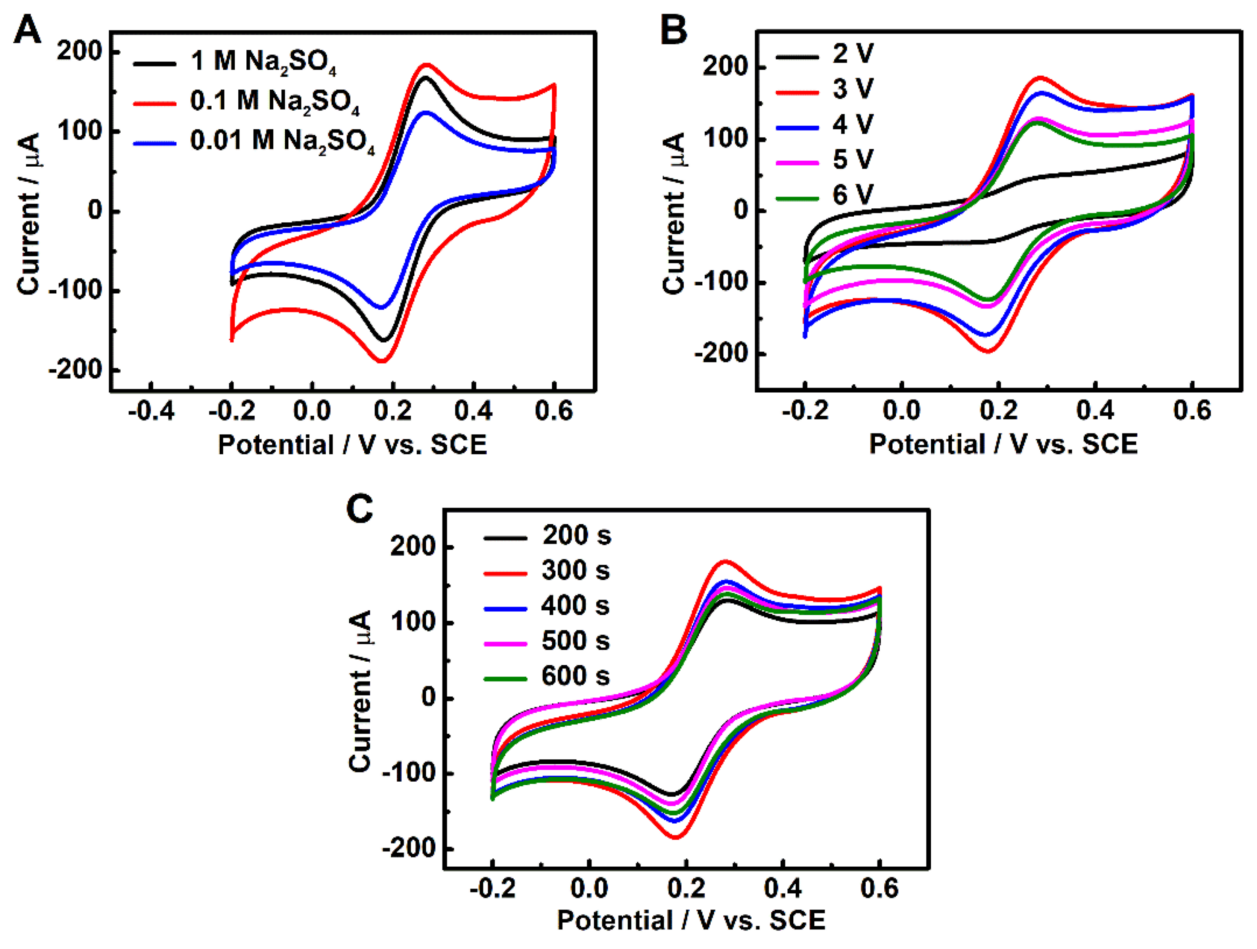
CV curves of 3DGrls/PGE prepared with different (**A**) electrolyte concentrations, (**B**) electrolysis potentials and (**C**) electrolysis times in a 5-mM [Fe(CN)_6_]^3−/4−^ solution at a scan rate of 50 mV s^−1^.

In order to investigate the effect of the electrolyte concentration on the performance of the modified electrode, Na_2_SO_4_ electrolytes with three different concentrations of 1 M, 0.1 M and 0.01 M were used to prepare 3DGrls/PGE. Figure 1A shows the cyclic voltammograms (CV) curves of the 3DGrls/PGE electrodes in 10 mL of 0.1-M KCl containing a 5-mM [Fe(CN)_6_]^3−/4−^ solution. It can be seen that the peak current increases with the increase of the concentration of Na_2_SO_4_ from 0.01 M to 0.1 M, indicating more ions available for graphite intercalation [42]. However, a decrease in the peak current can be observed when the concentration of Na_2_SO_4_ is as high as 1 M. A higher concentration of Na_2_SO_4_ means a lower amount of free water molecules left in the solution, resulting in a slower speed of water reduction at the cathode and insufficient hydroxyl ions (OH^−^) created. A lower concentration of OH^−^ ions leads to less attacked edge sites and grain boundaries of graphite, which leads to less formation of interconnected 3D graphene-like surfaces. Thus, the electrodes used in the subsequent study were all pretreated in 0.1-M Na_2_SO_4_ electrolyte.

#### 3.1.2. Electrolysis Potential

When a DC voltage is applied to the PGE electrode, the graphite layers of PGE will be expanded, and a spatial network of graphene will be created. Varied applied potentials lead to different degrees of a 3D-connected network of graphene and different electrochemical performances [43]. Figure 1B shows the CV curves of the electrodes obtained with different voltages applied in 10 mL of 5-mM [Fe(CN)_6_]^3−/4−^ solution. At a potential of 2 V, only a sluggish CV peak of [Fe(CN)_6_]^3−/4−^ with a big peak potential difference (ΔE_p_) can be observed, suggesting the unsuccessful creation of a 3D network on the surface of PGE because of the low voltage. When the voltage is increased to 3 V, the peak current dramatically increases. However, higher voltages result in a decrease in the peak current of [Fe(CN)_6_]^3−/4−^, which may be attributed to the dissociation and dispersion of graphite flakes into the electrolyte solution. Therefore, 3 V is the best electrolysis potential for preparing 3DGrls/PGE.

#### 3.1.3. Electrolysis Time

A varied electrolysis time will influence the electrochemical performance of the 3DGrls/PGE. A constant potential with time-varying electrolysis causes different degrees of graphite expansion and swelling, resulting in different surface areas, electrochemical conductivities and the mass transport of 3DGrls/PGE. As shown in Figure 1C, the highest CV response to [Fe(CN)_6_]^3−/4−^ is achieved at 300 s, and a longer time will decrease the current due to the overdissociation of graphite. Therefore, 300 s was selected for further studies.

### 3.2. Characterizations of 3DGrls/PGE 

**Figure 2:**
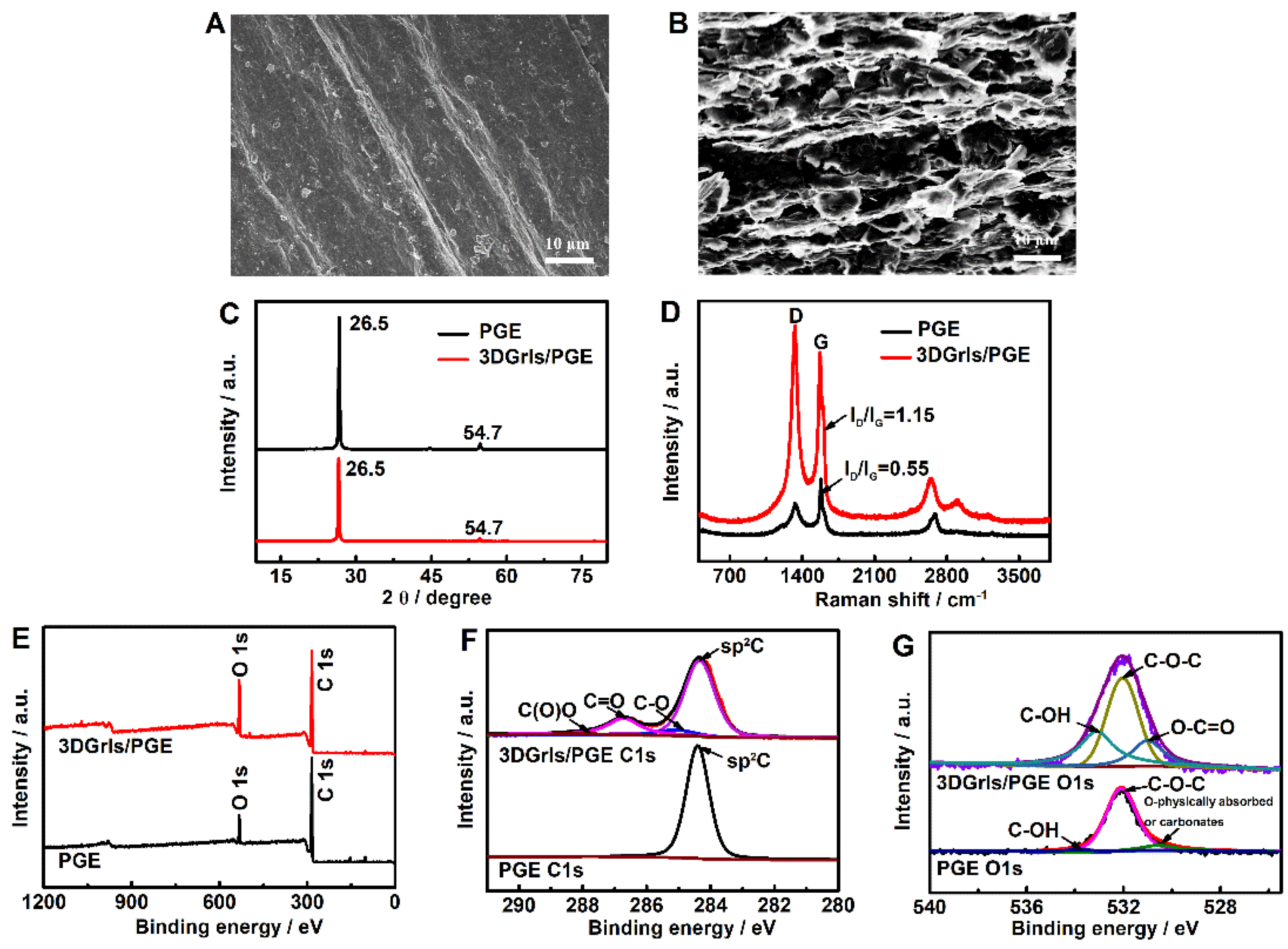
SEM images of (**A**) PGE and (**B**) 3DGrls/PGE. (**C**) XRD patterns and (**D**) Raman spectra of PGE and 3DGrls/PGE. (**E**) XPS full scan, (**F**) high-resolution C1 and (**G**) O1 spectra of PGE and 3DGrls/PGE.

The surface morphologies, crystal structures and chemical compositions of PGE and 3DGrls/PGE were characterized by SEM, XRD, XPS and Raman. Figure 2A,B shows the SEM images of PGE and 3DGrls/PGE. After the short time of electrolysis, the interconnected 3D scaffold structure can be clearly observed for 3DGrls/PGE. This porous network facilitates the penetration of the target molecules and provides a large electrochemically active surface area.

The XRD patterns of PGE and 3DGrls/PGE in Figure 2C reveal that both of them have two sharp diffraction peaks at 26.5° and 54.7° corresponding to the crystal plane of graphite (002) and (004), indicating that the crystal configuration of graphite is not changed by the electrolysis for 300 s at 3 V. The Raman spectra of PGE and 3DGrls/PGE in Figure 2D exhibit two characteristic peaks at 1331 cm^−1^ and 1572 cm^−1^ corresponding to the D-band and G-band of graphite. The D/G (I_D_/I_G_) intensity ratio of Raman spectroscopy is often used to appraise the sp^2^ ring clusters in sp^2^ and sp^3^-bonded carbon networks [47]. The I_D_/I_G_ value increases observably from PGE (0.55) to 3DGrls/PGE (1.15), indicating an increased average size of sp^3^ and more structural defects in 3DGrls/PGE because of the opening of the graphite edge layer after the electrochemical oxidation of the PGE surface [48].

In the full XPS spectra of PGE and 3DGrls/PGE (Figure 2E), although the two peaks of carbon and oxygen are both presented, the O/C ratio in 3DGrls/PGE is higher than that of PGE because of the electrolysis. The same results can be seen from the high-resolution C1s spectra (Figure 2F). Compared with the original PGE, 3DGrls/PGE demonstrates a much lower intensity of olefinic sp^2^ carbon, along with a higher oxygen content and sp^3^ hybrid carbon (C-O, C=O, O-C=O) [49]. As shown in the high-resolution O1s spectra (Figure 2G), three higher peaks can be observed at 531.31, 532.5 and 533.1 eV, corresponding to the O-C=O, C-O-C and C-OH groups of 3DGrls/PGE, respectively [50]. These results indicate that part of olefinic sp^2^ C of the original PGE is converted to sp^3^ C due to doping O during the electrochemical expansion process, which is consistent with the results of Raman spectroscopy [51].

### 3.3. Electrochemical Behaviors of 3DGrls/PGE

**Figure 3:**
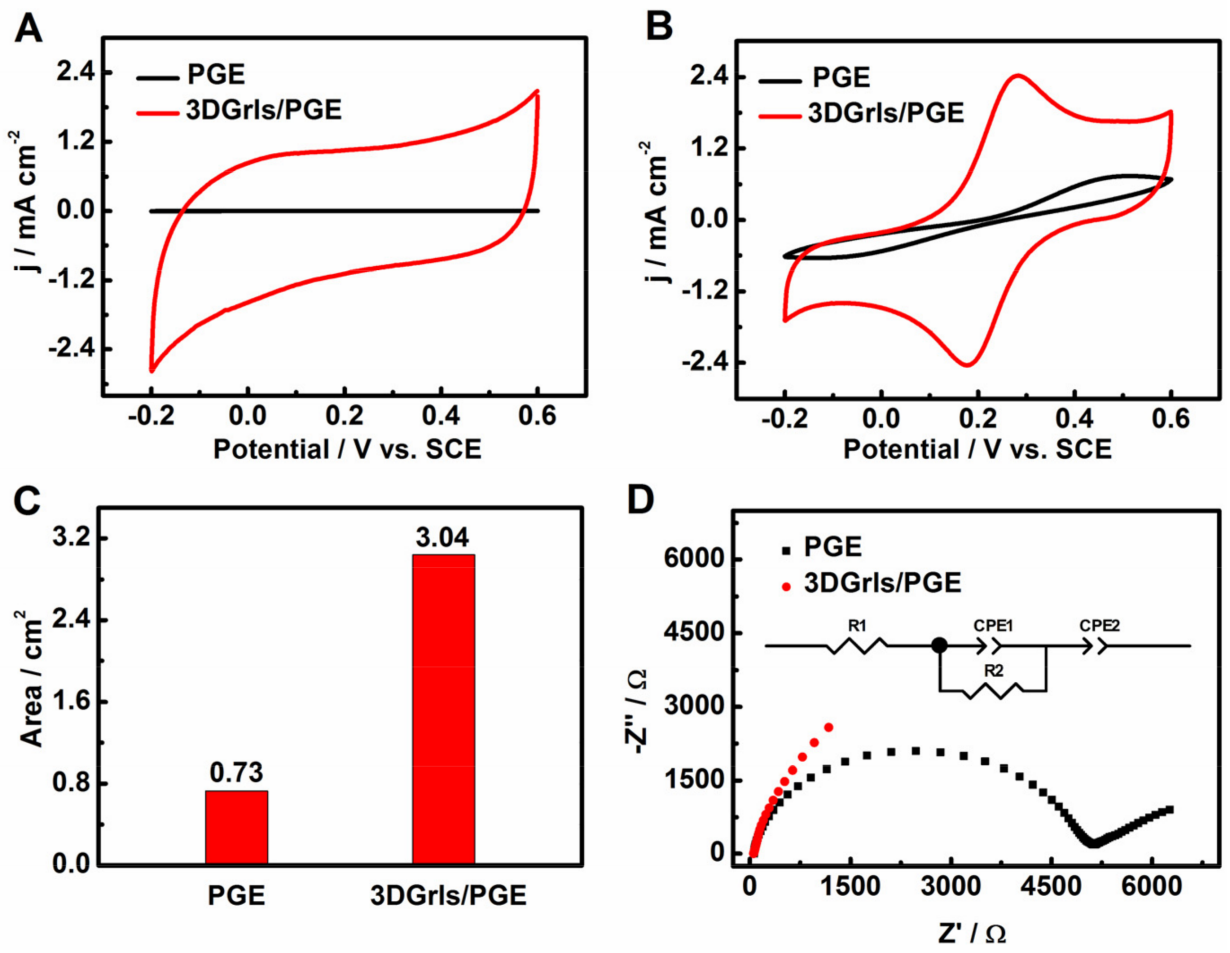
The CV curves of PGE and 3DGrls/PGE in (**A**) pH 9.18 PBS and (**B**) 0.1-M KCl solution containing 5-mM [Fe(CN)_6_]^3−/4−^ at a scan rate of 50 mV s^−1^. (**C**) The corresponding area histogram of PGE and 3DGrls/PGE. (**D**) The EIS of PGE and 3DGrls/PGE with the voltage amplitude at 50 mV in the frequency range 10^−2^–10^5^ Hz. The inset shows the respective equivalent circuit diagram, where R_1_, R_2_ and CPE represent the solution resistance, charge transfer resistance and constant phase elements, respectively.

To investigate the electrocatalytic behaviors of the 3DGrls/PGE, cyclic voltammograms were first performed in PBS and [Fe(CN)_6_]^3−/4−^ solutions. Compared with PGE, the background current of 3DGrls/PGE in PBS is greatly increased (Figure 3A), indicating a higher capacitance current due to the large specific surface area and pore structure of the 3D graphene-like surface. When 3DGrls/PGE was immersed in the [Fe(CN)_6_]^3−/4−^ solution, a pair of quasi-reversible redox peaks was observed (Figure 3B) with a smaller Δ*E*_p_ of 200 mV, while that at PGE was 500 mV, indicating that the 3D graphene-like surface on 3DGrls/PGE can promote electron transfer between the probes and 3DGrls/PGE [52]. Moreover, the peak current at 3DGrls/PGE was larger than that at PGE, indicating that 3DGrls/PGE had a larger electroactive surface area, which was proved by the determination results of the electroactive surface area of 3DGrls/PGE (3.04 cm^2^) and PGE (0.73 cm^2^) (Figure 3C). The EIS of 3DGrls/PGE (Figure 3D) shows a straight line, while that of PGE displays a depressed semi-circle. The charge transfer resistance (*R*_ct_) values of 3DGrls/PGE and PGE calculated from the equivalent circuit inset in Figure 3D are 363 and 4837 Ω, respectively. The dramatically decreased *R*_ct_ of 3DGrls/PGE exhibits lower electron transfer resistance and a greater electron transfer rate. The electrochemical behaviors of 3DGrls/PGE indicate that the 3D graphene-like surface formed on PGE within 5 min after constant electrochemical oxidation not only increases the specific surface area of the electrode but also accelerates the electron transfer rate.

### 3.4. Electrochemical Reduction of HMF at 3DGrls/PGE

**Figure 4:**
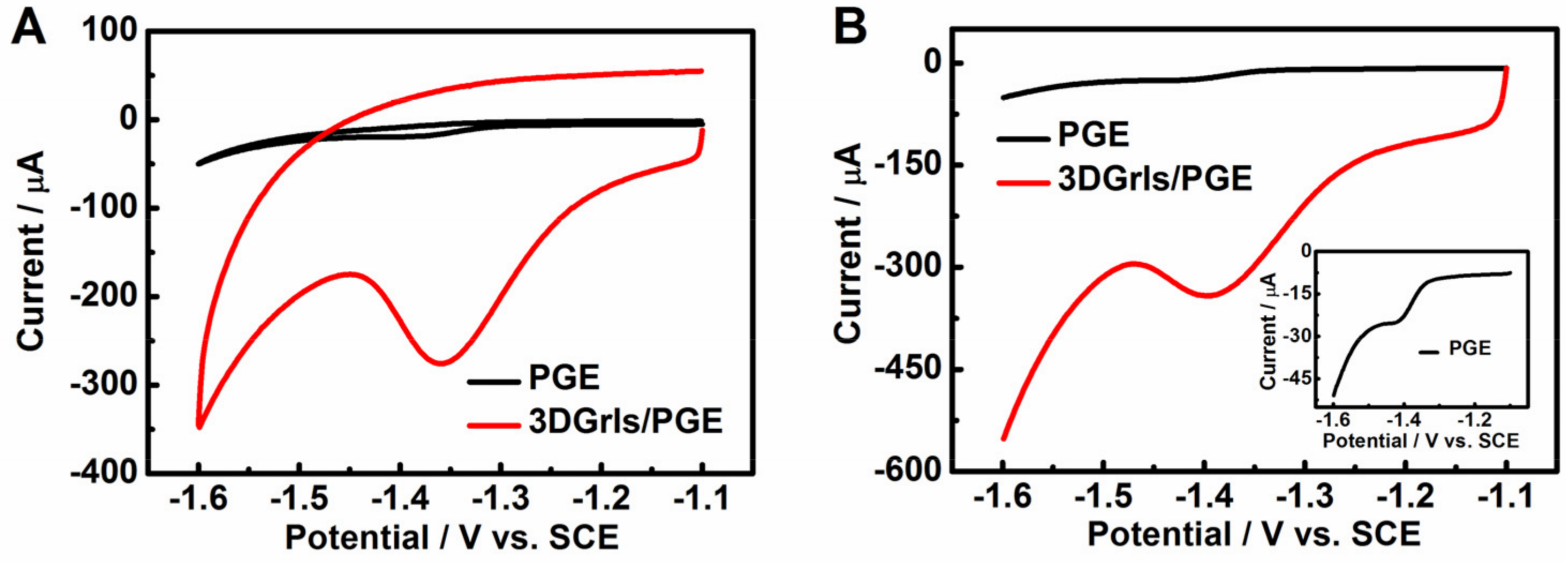
(**A**) CV and (**B**) LSV curves of PGE and 3DGrls/PGE in pH 9.18 PBS with 20-µM HMF. The inset is the LSV at PGE. Scan rate: 50 mV s^−1^.

The voltammetric performances of PGE and 3DGrls/PGE were further checked in PBS (pH 9.18) containing 0.5-mM HMF using CV and LSV with the curves shown in Figure 4. A reduction peak is presented at about −1.35 V, but no oxidation peak is observed for both CV curves (Figure 4A) in the potential range from −1.6 to −1.2 V, suggesting the irreversible electrochemical reduction of HMF at different electrodes. The reduction peak current (*I*_p_, *c*) of HMF at 3DGrls/PGE is 34 times that at PGE. A similar electrocatalytic activity for HMF is also found in the LSV results (Figure 4B), with a larger peak current (18 times higher) and a smaller (positive shift of 24 mV) overpotential at 3DGrls/PGE compared with that at PGE, indicating that 3DGrls/PGE has a better electrocatalytic activity for HMF reduction.

### 3.5. Effect of Scan Rate

**Figure 5:**
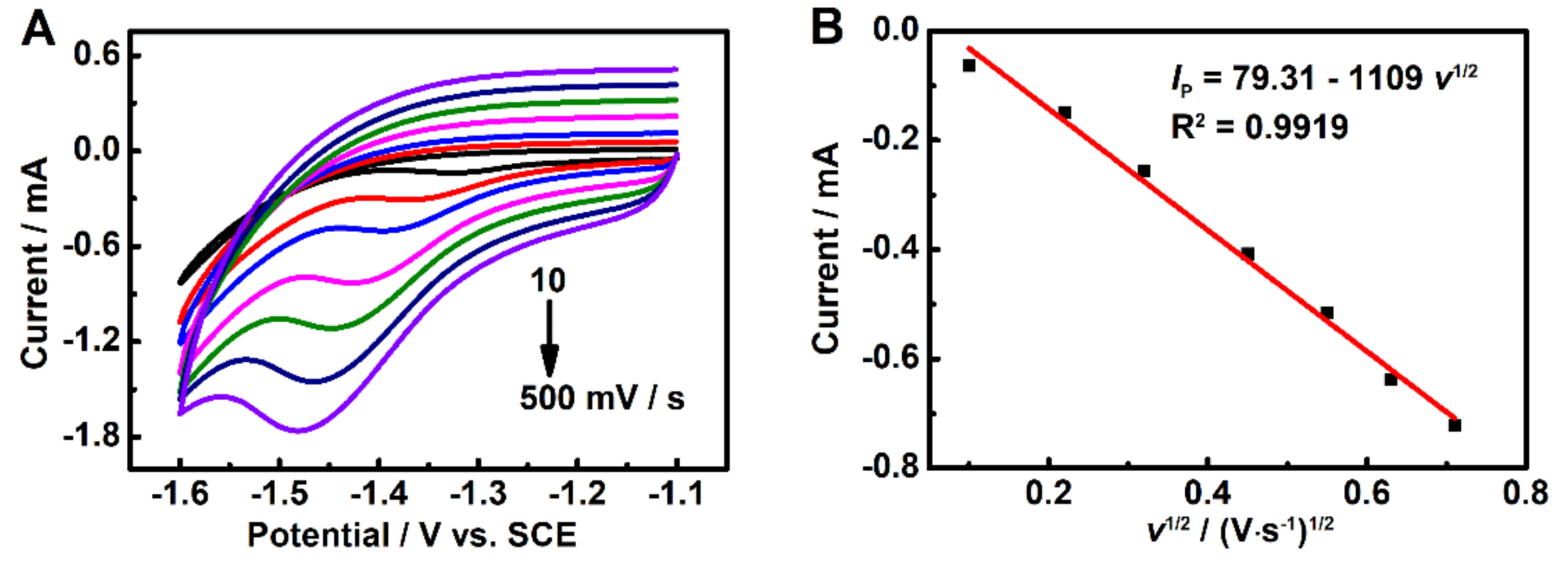
(**A**) CVs of 50-µM HMF in pH 9.18 PBS solution with scan rates from 10 to 500 mV s^−1^. (**B**) The linear relationship between the reduction peak current (*I*_p,c_) of HMF and the square root of the scan rate (*v*^1/2^).

**Scheme 1:**
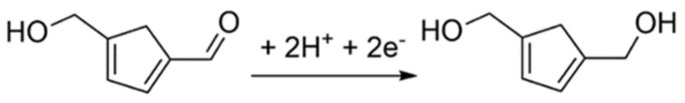
The electroreduction mechanism of HMF.

The cyclic voltammograms of HMF corresponding to various scan rates for the 3DGrls/PGE were investigated. As shown in Figure 5, the reduction peak current of HMF increases with the increase of the scan rate and is linear with the square root of the scan rate in the range of 10~500 mV s^−1^, while the reduction peak potential shifts negatively (Figure 5A). The regression equation between the *I*_p,c_ and the square root of the scan rate is as follows (Figure 5B): *I*_p_ = 79.31–1109 *v*^1/2^ (R^2^ = 0.9919), indicating a typical diffusion-controlled electrochemical process involving two electrons and two protons (Scheme 1) [30].

### 3.6. Determination of HMF

**Figure 6:**
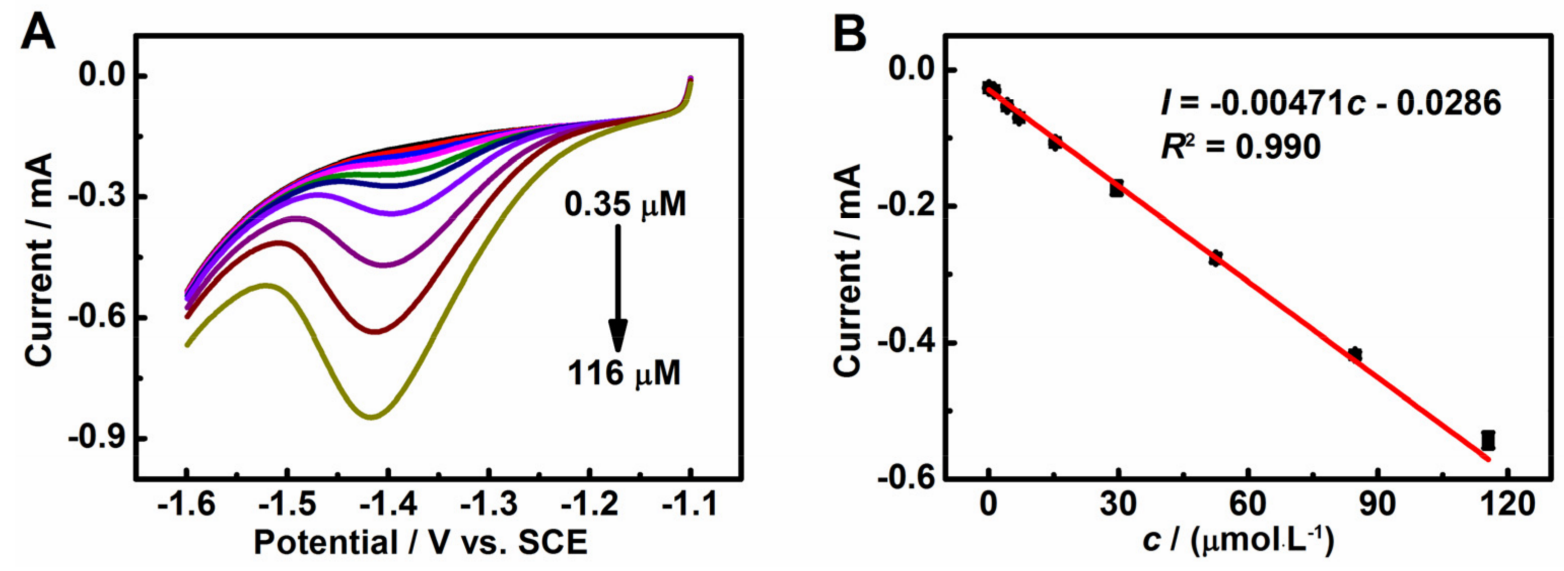
(**A**) LSV profiles at 3DGrls/PGE in pH 9.18 PBS containing different concentrations of HMF from 0.35 to 116 µM. (**B**) The relationship between the reduction peak current and HMF concentration. Scan rate: 100 mV s^−1^.

Figure 6A shows the LSV responses with different concentrations of HMF. As shown, the reduction peak current of HMF increases with the successive injection of HMF and displays a good linearity from 0.35 to 116 μM (Figure 6B), with a detection limit of 0.099 μM (0.012 μg/mL, S/N = 3) following the linear regression equation *I*_p_ = −0.00471*c* − 0.0286 (R^2^ = 0.990). The performance of 3DGrls/PGE was also compared with other modified electrodes, and the results are shown in Table 1. The 3DGrls/PGE with a wide linear range and a low detection limit can be used for the electrochemical quantitative determination of HMF in real samples.

### 3.7. Repeatability, Reproducibility, Stability and Selectivity of 3DGrls/PGE

**Figure 7:**
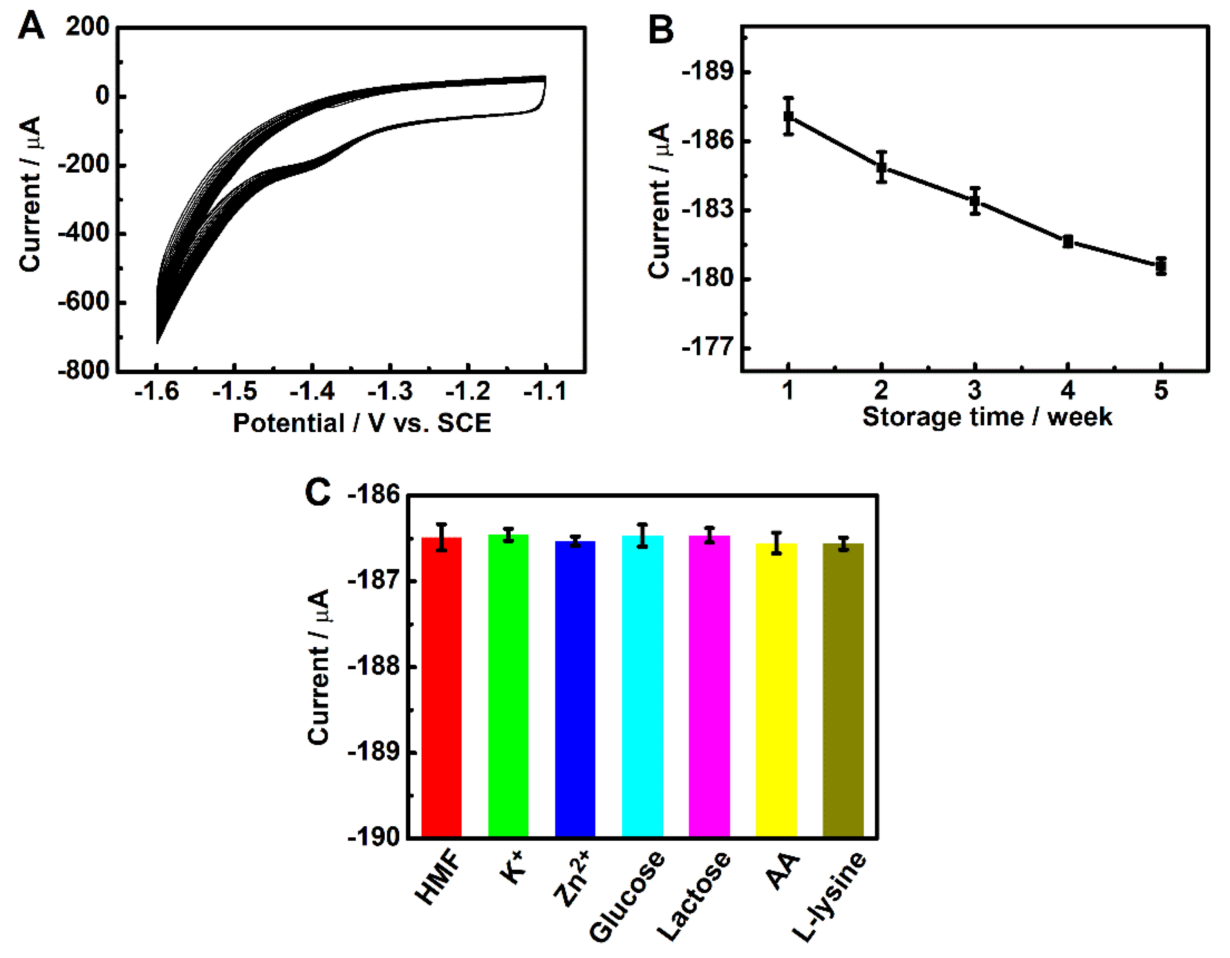
(**A**) CV curves of 100 consecutive scans of 50-µM HMF in pH 9.18 PBS solution at 3DGrls/PGE, and (**B**) the current responses after storage in a refrigerator for 1, 2, 3, 4 and 5 weeks. Scan rate: 50 mV s^−1^. (**C**) The reduction peak current in the presence of interfering substances.

The repeatability of the 3DGrls/PGE for the voltammetric response of 50-µM HMF was investigated by 100 successive measurements. It can be seen from Figure 7A that the corresponding reduction peak current of the 100th CV scan can still retain 78.7% of the original value, indicating that the 3DGrls/PGE has an excellent repeatability.

The reproducibility of the 3DGrls/PGE for the determination of 50-µM HMF in a pH 9.18 PBS was measured at three different parallel electrodes under the same conditions, respectively. The reduction peak currents of all the electrodes were almost the same, with a small relative standard deviation (RSD) of 4.5%, suggesting the 3DGrls/PGE has good reproducibility.

The long-term storage stability of 3DGrls/PGE was also evaluated by storing the same electrode for 5 weeks in a refrigerator. The corresponding reduction peak current after 5 weeks retained 96.3% of the original value, indicating that the 3DGrls/PGE with excellent storage stability can be used to detect HMF in real samples.

To evaluate the specificity of 3DGrls/PGE, possible interferences for the determination of 50-µM HMF in PBS (pH 9.18) were investigated by adding possible interferences into the processed cheese sample. It can be seen from Figure 7C that the concentrations of 100 times of K^+^ and Zn^2+^ and 50 times of lactose, glucose, L-lysine and AA cannot affect the determination of HMF with less than 6% change in the reduction peak current, indicating that 3DGrls/PGE has a wonderful anti-interference ability.

### 3.8. Real Sample Analysis

To evaluate the feasibility and validity of 3DGrls/PGE, the HMF amounts in three processed cheese samples were directly detected and compared with the HPLC results. As shown in Table 2, the developed method fits quite well with the HPLC method, indicating that the developed ultra-fast electrode is feasible for the determination of HMF in real samples.

## 4. Conclusions

A low-cost and pollution-free HMF-sensitive sensor was developed by applying constant potential oxidation on the PGE surface for 5 min. The obtained surface of the electrode is similar to 3D graphene. The thin graphene sheets are connected in space, and the electrode has a large specific surface area and a fast electron transfer rate. The results showed that the integrated 3DGrls/PGE has a good electrochemical response to HMF under optimal conditions. The linear range is wide (0.35~116 μM), and the detection limit is as low as 0.099 μM (S/N = 3). More importantly, the 3DGrls/PGE can be applied to determine the HMF in several processed cheese samples, and the results matched well with the HPLC results. The electrode preparation method proposed herein has the following advantages: (1) Simple and fast. A 3D graphene-like electrode surface can be generated in 300 s. (2) Green and friendly. No environmentally unfriendly reagents are required during the preparation process. (3) Good stability. The integrated electrode avoids the electrode material falling off the electrode surface and greatly improves the stability of the sensor.

## Figures and Tables

**Table 1:** Comparisons of the sensing performances of different electrodes for the determination of HMF.

Technique	Electrode	Real Sample	Linear Range (µg/mL)	LOD (µg/mL)	Ref.
SWV ^a^	SMDE ^d^	pharmaceutical and food	-	0.01	[25]
DPV	MDE ^e^	honey	-	0.048	[26]
PAD ^b^	NiNPs/GCE ^f^	sugarcane bagasse	0.1–1.26	0.054	[27]
CV and SWV	GCE	honey	0.15–1.26 (CV) 0.03–0.38 (SWV)	0.0833 (CV) 0.0072 (SWV)	[28]
LSV and CA ^c^	Cu-Ni MP/CuE ^g^	food	50.44–1261.10 (LSV) 0.05–1387.21 (CA)	0.443 (LSV) 0.005 (CA)	[29]
DPV	BP-GCE ^h^	milk	0.1–10	0.012	[30]
LSV	3DGrls/PGE	processed cheese	0.044–14.63	0.012	This work

^a^ Square wave voltammetry. ^b^ Pulsed amperometric detection. ^c^ Chronoamperometry. ^d^ Static mercury drop electrode. ^e^ Mercury drop electrode. ^f^ Glassy carbon electrode chemically modified with nickel nanoparticles. ^g^ Cooper electrode modified with Cu-Ni bimetallic microparticles. ^h^ Black phosphorene-modified GCE.

**Table 2:** Determination of HMF in real samples with the 3DGrls/PGE (*n* = 5).

Sample	3DGrls/PGE		HPLC	
	HMF (μg/g)	RSD (%)	HMF (μg/g)	RSD (%)
Processed cheese A	86.84	5.2	83.55	2.1
Processed cheese B	82.01	2.9	80.66	1.8
Processed cheese C	75.37	3.9	76.88	1.5

## Data Availability

The data is contained within the article.
